# A Generalization of the DMC †

**DOI:** 10.3390/e28020228

**Published:** 2026-02-16

**Authors:** Sergey Tridenski, Anelia Somekh-Baruch

**Affiliations:** Faculty of Engineering, Bar-Ilan University, Ramat Gan 5290002, Israel

**Keywords:** error exponent, correct-decoding, discrete memoryless channel capacity

## Abstract

We consider a generalization of the discrete memoryless channel, in which the channel probability distribution is replaced by a uniform distribution over clouds of channel output sequences. For a random ensemble of such channels, we derive an achievable error exponent, as well as its converse together with the optimal correct-decoding exponent, all as functions of information rate. As a corollary of these results, we obtain the channel ensemble capacity.

## 1. Introduction

We consider the basic information-theoretic scenario of point-to-point communication. The standard go-to model for such a scenario is the discrete memoryless channel (DMC). With this model, the communication performance is characterized by the channel capacity, surrounded by the error and the correct-decoding exponents, as functions of the information rate. In order to be characterized by these quantities, the communication is usually performed with a codebook of blocks of *n* channel input symbols, conveying $2^{nR}$ equiprobable messages, where *R* is the rate in bits.

In this paper, we slightly deviate from the standard DMC model. In our set-up, the DMC itself reappears as a limiting case. Consider first the following communication scheme. Let *K* be some positive real parameter in addition to the rate *R*, and suppose that there has been an exponentially large number $2^{n(R\;+\;K)}$ of block transmissions through a DMC. Each transmitted block is a codeword of length *n*, chosen each time with uniform probability from the same codebook of size $2^{nR}$. This corresponds to a significant amount of transmitted information of $nR\cdot 2^{n(R\;+\;K)}$ bits. By the end of these transmissions, each of the $2^{nR}$ codewords has been used approximately $2^{nK}$ times, resulting in $2^{nK}$ not necessarily distinct channel output vectors, forming an unordered “cloud”. The parameter *K* therefore represents an exponential size of the cloud of channel output vectors generated by a single codeword. Suppose that, in the end of the $2^{n(R\;+\;K)}$ transmissions, the unordered outcome clouds of all the codewords are revealed to the decoder. For small *K*, when most of the output vectors in the clouds are distinct, this “revelation” would be approximately equivalent to a noiseless transmission of the same $nR\cdot 2^{n(R\;+\;K)}$ bits of information. For higher *K*, however, the description of the clouds will require an exponentially smaller number of noiseless bits compared to $nR\cdot 2^{n(R\;+\;K)}$.

Note that, given the $2^{n(R\;+\;K)}$ received channel output blocks with time indices $j=1,\ldots ,2^{n(R\;+\;K)}$, as well as the knowledge of the clouds, the optimal decoder for any given output block with an index *j* (optimal in the sense that it minimizes the probability of error for the block with the index *j*) chooses the codeword with the maximal number of replicas of this block in its cloud. This decoder is optimal regardless of the message probabilities or the transition probabilities of the DMC that created the clouds. Moreover, the same decoder, which relies on the clouds and is oblivious of the transition probabilities in the channel that created the clouds, remains optimal whether or not the channel is memoryless or time-invariant within each block, as long as it is memoryless and time-invariant by blocks.

As an alternative communication scheme, consider $2^{n(R\;+\;K)}$ contemporaneous block transmissions to $2^{n(R\;+\;K)}$ receivers through physically distinct channels, modeled as stochastically independent and identical DMCs. Each transmitted block is a codeword of length *n*, chosen independently of others with uniform probability from the same common codebook of size $2^{nR}$. Suppose that, with noiseless feedback, all the $2^{n(R\;+\;K)}$ received channel output vectors become associated with the respective sent codewords on the side of the transmitters and then, with cooperation between the transmitters, this information becomes available (published) to the receivers in the form of $2^{nR}$ unordered outcome clouds of the average size of $2^{nK}$ channel output vectors associated with the codewords, as in the previous scheme. This can be seen as a joint estimation of physically distinct but stochastically identical channels. As soon as its received vector is published, a receiver can start decoding. Our current work shows that the smaller the clouds, the lower the average probability of error and the higher the capacity of the resulting channel.

Given the clouds, the receiver sees effectively a different channel—one that chooses its output vector with uniform probability from the cloud of the sent codeword. This channel can be described by a model, different from DMC. In this model, we assume that the messages are equiprobable and each cloud contains exactly $2^{nK}$ vectors. The clouds are generated randomly i.i.d. with a channel-generating distribution, independently for each codeword in a codebook. This is similar to constant composition clouds used for superposition coding [1] through a noiseless channel. The capacity and the relevant probability exponents of this scheme can be given in the average sense, for the ensemble of random channels. As the exponential size of the clouds *K* tends to infinity, the random channel ensemble converges to a single channel with the transition probabilities of the channel-generating distribution, which is a DMC in our case [2,3,4].

In this paper, we complete our work [5]. We make a rigorous proof of the random-coding error exponent [5] [Theorem 1] and add an error exponent converse bound. We verify that the correct-decoding exponent converse [5] [Theorem 2] is achievable.

The paper is organized as follows. In Section 2, we start introducing our notation and define the channel model. In Section 3, we derive an achievable error exponent for the random channel ensemble. In Section 4 and Section 5, we provide converse results. We derive an upper bound on the optimal error exponent (in Section 4) and the optimal correct-decoding exponent (in Section 4 and Section 7) of the random channel ensemble. In Section 6, we obtain the channel ensemble capacity as a corollary of the previous sections.

## 2. Channel Model

Let x∈X and y∈Y be letters from finite channel input and output alphabets, respectively. Let W(y|x) denote transition probabilities of a channel-*generating* distribution. The channel is generated for a given codebook of blocks of a length *n* of letters from X. Let C(n be such a codebook, consisting of $\left\lfloor e^{nR}\right\rfloor$ codewords x(m∈X(n, $m=1,2,\ldots ,\lfloor e^{nR}\rfloor$, where *R* is a positive real number representing a communication rate.

Given this codebook and another positive real number *K*, a channel *instance* is generated with the distribution *W*, as follows. For each one of the $\left\lfloor e^{nR}\right\rfloor$ messages *m*, an exponentially large number $\left\lfloor e^{nK}\right\rfloor$ of sequences y∈Y(n is generated randomly given the corresponding codeword x(m, where each sequence y is generated independently of others. Each letter y(i, i=1,2,…,n, of each such sequence y is generated i.i.d. according to *W* given the corresponding letter x(mi of x(m. In this way, the set of clouds of y’s of size $\left\lfloor e^{nK}\right\rfloor$ for each *m* forms one channel instance.

We assume that the messages $m=1,2,\ldots ,\lfloor e^{nR}\rfloor$, represented by the codewords x(m, are equiprobable. Given that a particular message is sent through the channel, the stochastic channel *action* now amounts to choosing exactly one of all the not necessarily unique $\left\lfloor e^{nK}\right\rfloor$ vectors y, corresponding to the sent message, with the uniform probability $1/\lfloor e^{nK}\rfloor$. We assume that the decoder, receiving the channel output vector $\hat{y}$, knows not only the codebook, but also the channel instance, i.e., all the $\left\lfloor e^{nR}\right\rfloor$ clouds comprising the corresponding y’s.

A cloud can have more than one replica of the received vector $\hat{y}$. The maximum-likelihood (ML) decoder makes an error with non-vanishing probability $\geq \frac{1}{2}$, if there exists an incorrect message with the same or a higher number of replicas of $\hat{y}$ in its cloud, comparing to the sent message itself. Otherwise, there is no error.

Let y(m,ℓ with indices $m=1,2,\ldots ,\lfloor e^{nR}\rfloor$ and $\ell =1,2,\ldots ,\lfloor e^{nK}\rfloor$ be all the cloud vectors, not necessarily distinct. Then, the encoder is a function $f:\left\{1,\ldots ,\lfloor e^{nR}\rfloor \right\}\to \left\{1,\ldots ,\lfloor e^{nR}\rfloor \right\}$, mapping the messages to the clouds, which is f(m)=m, ∀m. The ML decoder without loss of optimality is a deterministic function g:R(n→1,…,⌊enR⌋, given by

g(y)=minargmax1≤m≤⌊enR⌋∑ℓ=1⌊enK⌋1{y(m,ℓ=y},

where the minimum is taken over the indices *m* in the argmax set.

## 3. Achievable Error Exponent

Suppose the codebook is generated i.i.d. according to a distribution over X with probabilities P(x). Let P(¯(e(n) denote the average error probability of the maximum-likelihood decoder, averaged over all possible messages, codebooks, and channel instances:

(1) P¯e(n)≜1⌊enR⌋∑i=1⌊enR⌋E(PWPrg(Y)≠I|Y(m,ℓℓ=1⌊enK⌋,X(mm=1⌊enR⌋,I=i,

where Y represents the random received vector, while *I* is the random sent message, X(m are randomly generated codewords, Y(m,ℓ are the random cloud vectors, and the expectation E(PW is taken according to the independent and identical joint distribution PW. Let T(y)V(x|y) denote probabilities in an auxiliary distribution over X×Y and let us define the following:

ATVP≜D(V∥P|T),HT≜E(T−logT(Y),

(2) B(TVW≜E(TV−logW(Y|X),

(3) E(e(P,R,K)≜minTVD(TV∥PW)+|A(TVP−R+|B(TVW−K|+|+,

where $D\left(V\;∥\;P\;|\;T\right)=\sum_{x,\;y}T\left(y\right)V\left(x\;|\;y\right)\log\frac{V(x\;|\;y)}{P(x)}$ is the Kullback–Leibler divergence averaged over *T*, the expectation E(TV is with respect to the joint distribution TV, and ${\;|\;t\;|}^{+}≜\max\;\left\{0,t\right\}$. All the logarithms here and below are to base *e*. In what follows, we usually suppress the superscripts in A(TV and B(TV. Then, we can show the following:

**Theorem 1** (Random-coding error exponent)**.** 
(4) limn→∞−1nlogP¯e(n)=E(e(P,R,K),

*where E(e(P,R,K) is defined in (3).*

We prove this theorem by separately deriving matching lower and upper bounds. For the lower bound, for ϵ∈R, let us further define

(5) E(e(P,R,K,ϵ)≜minE(1(P,R,K,ϵ),E(2(P,R,K,ϵ),

(6) E(1(P,R,K,ϵ)≜minTVV˜:B(TV˜≤B(TV+ϵ≤KF(TV,TV˜),

(7) E(2(P,R,K,ϵ)≜minTVV˜:B(TV+ϵ≥KF(TV,TV˜),

(8) F(TV,TV˜)≜D(TV∥PW)+|A(TV˜−R+|B(TV˜−K|+|+,

where A(TV˜ and B(TV are defined by (2). Our lower bound is given by Lemma 1, together with Lemmas 3 and 4 below.

**Lemma 1** (Lower bound)**.** 
(9) lim infn→∞−1nlogP¯e(n)≥limϵ↘0(+E(e(P,R,K,ϵ),

*where E(e(P,R,K,ϵ) is defined in (5)–(8).*

In the proof of Lemma 1, we use the following auxiliary lemma:

**Lemma 2** (Super-exponential bounds).  *Let Z(i*, *i=1*, 2,…, *be i.i.d. Bernoulli($e^{-nB}$) random variables. Then, for any δ>0*,

(10) Pr∑i=1⌊enK⌋Z(i≥en|K−B|++δ≤exp−en|K−B|++δ+o(1),

(11) Pr∑i=1⌊enK⌋Z(i≤enK−B−δ≤exp−enK−B+o(1),

*where o(1) is a function of (δ,K) that satisfies o(1)→0 as n→∞.*

**Proof.**  The result follows straightforwardly from Markov’s inequality for the random variable e∑iZ(i and e−∑iZ(i, resp., as well as the inequality $1+t\leq e^{t}$. □

**Proof of Lemma 1.**  We will use ϵ>0 to establish (9).

Let x be the sent codeword and $\hat{y}$ be the received vector. The cloud of x can contain more than one vector $\hat{y}$. The maximum-likelihood decoder makes an error with non-vanishing probability $\geq \frac{1}{2}$; if there exists an incorrect codeword (not necessarily distinct from x, but representing a different message and having therefore an independently generated cloud) with the same or a higher number of vectors $\hat{y}$ in its cloud, compared to the sent codeword itself. Otherwise, there is no error.

Consider an event where x and $\hat{y}$ have a joint empirical distribution (type with denominator *n*) TV, i.e., TV∈P(n(Y×X), where *T* is a distribution on Y and *V* is a conditional distribution on X given a letter from Y. The exponent of the probability of this event (probability of type class in [6]) is given by

D(TV∥PW)+o(1),

where the term o(1) vanishes uniformly w.r.t. TV, as n→∞.

Consider now the competing codewords. The exponent of the probability of an event, in which $\hat{y}$ appears somewhere in the clouds corresponding to the incorrect codewords, is given by

(12) minV˜:TV˜∈P(n(Y×X)|A(TV˜−R+|B(TV˜−K|+|++o(1),

where o(1) is uniform w.r.t. *T*. To observe this, consider a possibly different (from *V*) conditional type $\tilde{V}$ of some $\tilde{x}$ w.r.t. $\hat{y}$. The exponent of the probability of an event, in which a certain incorrect codeword belongs to the conditional type $\tilde{V}$ given $\hat{y}$, is given by

(13) A(TV˜+o(1),

where o(1) is uniform w.r.t. $T\tilde{V}$. The exponent of the probability of an event, that a certain y in the cloud of $\tilde{x}$ equals $\hat{y}$, is given by B(TV˜. The exponent of the probability of an event, that in the cloud of $\tilde{x}$ of the type $\tilde{V}$ the vector $\hat{y}$ appears at least once, is given by

(14) |B(TV˜−K|++o(1),

where the term o(1), vanishing as n→∞, depends on *K*. In particular, as a *lower* bound on the exponent, (14) follows trivially without o(1) from the union bound on the probability. Meanwhile, to confirm (14) as an *upper* bound on the exponent, denoting e−nB(TV˜≜α and $\left\lfloor e^{nK}\right\rfloor ≜M\equiv \beta^{-1}$, we can write similarly to [3] [Equation (14)]:

Pry^isinthecloudofx˜=1−(1−α)M≡α∑j=0M−1(1−α)(j≥min{α,β}∑j=0M−1(1−β)(j≡min{αβ−1,1}[1−(1−β)M︸≤1/e]=e−n(|BTV˜−K|++o(1)),

where o(1) is a function of *K*. Adding together (13) and (14), we obtain the exponent of the probability of an event, that a certain incorrect codeword is of the conditional type $\tilde{V}$ w.r.t. $\hat{y}$, *and* $\hat{y}$ appears at least once in its cloud:

(15) A(TV˜+|B(TV˜−K|++o(1),

where o(1) is uniform w.r.t. $T\tilde{V}$. Finally, the exponent of the probability of an event, where there exists in the codebook at least one incorrect codeword of the conditional type $\tilde{V}$ w.r.t. $\hat{y}$ and $\hat{y}$ appears at least once in its cloud, is given by

(16) |A(TV˜+|B(TV˜−K|+−R|++o(1),

where o(1)→0 uniformly w.r.t. $T\tilde{V}$ as n→∞ and may depend on *K* and *R*, which yields (12).

Suppose that K−B(TV≤ϵ. In this case, the exponent of the conditional probability of error, given that the received vector and the sent codeword belong to the joint type, TV can be lower bounded by (12), and the exponent of the (unconditional) probability of error due to all such cases is lower-bounded by

(17) minTVV˜:TV,TV˜∈P(n(Y×X),K−B(TV≤ϵD(TV∥PW)+|A(TV˜−R+|B(TV˜−K|+|++o(1).

Consider now the opposite case when K−B(TV≥ϵ. For this case, recall that the exponent of the probability of an event, in which there exists at least one incorrect codeword of the conditional type $\tilde{V}$ w.r.t. $\hat{y}$, is given by |A(TV˜−R|++o(1). Suppose now that the conditional type $\tilde{V}$ is such that K−B(TV˜≤K−B(TV−ϵ. For this case, we use Lemma 2, with δ=ϵ/2. Using (11) for the correct cloud and (10) for the competing clouds, the probability of the event that the cloud of an incorrect codeword of the type $\tilde{V}$ has at least as many occurrences of the vector $\hat{y}$, compared to the correct codeword of the type *V*, can be upper-bounded uniformly by

(18) $$\underset{\mathrm{the}\;\mathrm{correct}\;\mathrm{cloud}}{\underset{︸}{\exp\left\{\;-e^{n(\epsilon +o(1))}\right\}}}\;+\;\underset{\mathrm{the}\;\mathrm{competing}\;\mathrm{clouds}}{\underset{︸}{\exp\left\{nR-e^{n(\epsilon /2+o(1))}\right\}}}.$$

That is, it tends to zero *super*-exponentially fast with *n*. The remaining types $\tilde{V}$ with K−B(TV˜≥K−B(TV−ϵ allow us to write a lower bound on the exponent of the (unconditional) probability of error due to all the cases K−B(TV≥ϵ, as

(19) minTVV˜:TV,TV˜∈P(n(Y×X),K−B(TV˜≥K−B(TV−ϵ≥0D(TV∥PW)+|A(TV˜−R|++o(1).

Together, (17) and (19) cover all cases and the minimum between the two gives the lower bound on the error exponent.

Observe that the objective function of (17) can also be used in (19), because in (19), the set over which the minimization is performed satisfies B(TV˜≤K. Furthermore, for the lower bound, we can simply extend the minimization set in (17) and (19) from types to arbitrary distributions TV and $T\tilde{V}$. Therefore, omitting o(1), in the limit of a large *n*, we can replace the minimum of the bounds (17) and (19) with (5). □

To complete the lower bound given by Lemma 1, we establish the next two lemmas.

**Lemma 3** (Epsilon equals zero)**.**  *The expression defined in (5)–(8) satisfies*

(20) limϵ↘0(+E(e(P,R,K,ϵ)=E(e(P,R,K,0).

**Proof.**  Observe first that both (6) and (7) are convex (∪) functions of ϵ∈R. This can be verified directly by the definition of convexity, using the property that $F(TV,\;T\tilde{V})$ is convex (∪) and B(TV is linear in the pair $\left(TV,\;T\tilde{V}\right)$. Furthermore, by continuity of $F(TV,\;T\tilde{V})$ and B(TV, it follows that (6) and (7) are *lower semi-continuous* functions of ϵ∈R. Observe next from (6) and (7) that at least one of them is necessarily finite at ϵ=0, i.e., E(e(P,R,K,0)<+∞. Suppose that E(2(P,R,K,0)≤E(1(P,R,K,0). Then, E(2(P,R,K,ϵ) is finite for ϵ≥0 and by the lower semi-continuity of the convex function limϵ↘0E(2(P,R,K,ϵ)=E(e(P,R,K,0). Then, we also obtain (20). Consider the opposite case E(1(P,R,K,0)<E(2(P,R,K,0). Then, (6) at ϵ=0 is a minimization of a continuous function of $TV\tilde{V}$ over a closed non-empty set. Let $\hat{T}\hat{V}$ be the distribution TV, achieving the minimum in (6) at ϵ=0. Then, necessarily K>B(T^V^ (otherwise with K=B(T^V^ there has to be E(1(P,R,K,0)≥E(2(P,R,K,0)). Then, E(1(P,R,K,ϵ) is finite for K−B(T^V^>ϵ≥0 and by the lower semi-continuity of the convex function limϵ↘0E(1(P,R,K,ϵ)=E(e(P,R,K,0). Then, we once again obtain (20). □

**Lemma 4** (Identity)**.** 
(21) E(e(P,R,K,0)≡E(e(P,R,K),

*where the LHS and the RHS are defined by (5)–(8) and (3), respectively.*

**Proof.**  For ϵ=0, we can conveniently rewrite the minimum (5) between (6) and (7) in the following unified manner:

(22) E(e(P,R,K,0)=minTVV˜:K−B(TV≤|K−B(TV˜|(+{−H(T+A(TV+B(TV︸=D(TV∥PW)+|A(TV˜+B(TV˜+|K−B(TV˜|+−K︸=|BTV˜−K|+−R|+},

where in the objective function we used also the property that ${\left|\;t\;\right|}^{+}=t+{\left|-t\;\right|}^{+}$. Now, it is convenient to verify, that in (22) the conditional distribution $\tilde{V}$ without loss of optimality can be replaced with *V*. To this end suppose that some joint distributions TV and $T\tilde{V}$ satisfy the condition under the minimum of (22).

If A(TV+B(TV≤A(TV˜+B(TV˜, then, since also |K−B(TV|+≤|K−B(TV˜|+, we cannot increase the objective function of (22) by using TV in place of $T\tilde{V}$.

If A(TV+B(TV≥A(TV˜+B(TV˜, then we cannot increase the objective function of (22) by using $T\tilde{V}$ in place of TV.

It follows that (3) is a lower bound on minimum (22). Finally, since (3) is also an upper bound on (22), we conclude that there is equality between (3) and (22). □

Combining (21), (20), and (9), we have that the RHS of (4) is a lower bound. It remains to show that it is also an upper bound.

**Lemma 5** (Upper bound)**.** 
(23) lim supn→∞−1nlogP¯e(n)≤E(e(P,R,K),

*where E(e(P,R,K) is defined in (3).*

In the proof of Lemma 5 we use the following auxiliary lemma:

**Lemma 6** (Two competing clouds)**.**  *Let N∼B(M,α) and N(1∼B(M−1,α) be two statistically independent binomial random variables with the parameters M≥2 and α∈(0,q]∪{1}, where q∈(0,1) is a constant. Then,*

(24) PrN≥N(1+1|N≥1≥121−1/2π+o(M(1),

*where o(M(1) depends on q and as M→+∞ satisfies o(M(1)→0.*

The proof is given in the Appendix A. In the above Lemma, *N* and N(1+1 can describe the random numbers of replicas of $\hat{y}$ in an incorrect cloud and the correct cloud, respectively.

**Proof of Lemma 5.**  For the upper bound it is enough to consider the exponent of the probability of the event that the transmitted and the received blocks x and $\hat{y}$ have a joint type TV, while in the codebook there exists at least one incorrect codeword of the *same* conditional type *V* w.r.t. $\hat{y}$, and $\hat{y}$ appears at least once in its cloud. As in the proof of Lemma 1, this exponent is given by

(25) D(TV∥PW)+|A(TV−R+|B(TV−K|+|++o(1).

The additional exponent of the conditional probability of error given this event is o(1), as follows immediately by Lemma 6, used with $M=\lfloor e^{nK}\rfloor$ and α=e−nB(TV with $q=\max_{\;W(y\;|\;x)\;<\;1}W\left(y\;|\;x\right)>0$, or α=1. In the limit of a large *n*, we can omit o(1) and by continuity minimize (25) over all distributions TV, to obtain the RHS of (23). □

This completes the proof of Theorem 1. An alternative representation of the error exponent of Theorem 1 is given by

**Lemma 7** (Dual form)**.**
(26) E(e(P,R,K)=sup0≤η≤ρ≤1E(0(ρ,η,P)−ρR−ηK,

*where E(e(P,R,K) is defined in (3) and*

(27) E(0(ρ,η,P)≜−log∑y[∑xP(x)W1+η1+ρ(y|x)]1+ρ.

**Proof.**  Observe first that the minimum (3) can be lower-bounded as

(28) minTVD(TV∥PW)+|A(TV−R+|B(TV−K|+|+≥sup0≤ρ≤1

(29) minTVD(TV∥PW)+A(TV−R+|B(TV−K|+·ρ.

Observe further, that the lower bound (29) is the *lower convex envelope* of (28) as a function of R∈R. Indeed, the minimum (28) is a non-increasing function of *R*, and therefore it cannot have *lower supporting lines* with slopes greater than 0. It also cannot have lower supporting lines with negative slopes below −1, as it decreases with the slope exactly −1 in the region of negative or small positive values of *R*. Note that the objective function of the minimum (29) is continuous in TV in the closed region of TV≪PW. Let $T_{\;\rho }\;V_{\;\rho }$ be the minimizing distribution of the minimum in (29) for a given ρ∈[0,1]. For this distribution, there exists a real R=R(ρ) such that the expression in the square brackets of (29) is zero. Therefore, there is equality between (29) and (28) at R(ρ). And this is achieved for each ρ∈[0,1], which corresponds to lower supporting lines of slopes −ρ between 0 and −1.

Finally, we observe that there is in fact equality between (28) and (29) for all *R*, since (28) is a convex (∪) function of *R* and, therefore, it coincides with its lower convex envelope. Indeed, using the property ${\left|\;t\;\right|}^{+}=\max\;\left\{0,t\right\}$, the objective function of the minimization (28) can be rewritten as a maximum of three terms:

max{D(TV∥PW),D(TV∥PW)+A(TV−R,D(TV∥PW)+A(TV−R+B(TV−K}.

Then, this objective function is convex (∪) in the triple (TV,R,K), verified as a maximum of convex (∪) functions of (TV,R,K). In particular, the convexity of A(TV=D(V∥P|T)≡D(TV∥TP) in TV follows by the log-sum inequality [6]. By the definition of convexity, it is then verified that the minimum (28) itself is a convex (∪) function of *R*.

So far, we have shown that (28) and (29) are equal. Consider now the minimum of (29) with any ρ∈[0,1]:

(30) minTVD(TV∥PW)+ρA(TV−R+|B(TV−K|+≥sup0≤β≤1

(31) minTVD(TV∥PW)+ρA(TV−R+B(TV−K·β.

By the same reasoning as before, there is equality also between (30) and (31). Putting together (31) and (29) and denoting β·ρ=η, we can rewrite (28) as

sup0≤ρ≤10≤η≤ρminTVD(TV∥PW)+ρA(TV−R+ηB(TV−K=sup0≤ρ≤10≤η≤ρminTV{D(T∥Tρ,η)+(1+ρ)D(V∥Vρ,η|T)︸≥0+E(0(ρ,η,P)−ρR−ηK},

where the minimizing solution is

(32) $$T_{\;\rho ,\;\eta }\left(y\right)V_{\;\rho ,\;\eta }\left(x\;|\;y\right)\;\equiv \;\frac{1}{c}\cdot P\left(x\right)W^{\frac{1+\eta }{1+\rho }}\left(y\;|\;x\right){\left[\sum_{\tilde{x}}P\left(\tilde{x}\right)W^{\frac{1+\eta }{1+\rho }}\left(y\;|\;\tilde{x}\right)\right]}^{\rho }.$$

□

## 4. A Converse Theorem for the Error and Correct-Decoding Exponents

Let P(e(C(n) denote the average error probability of the maximum-likelihood decoder for a given codebook C(n of block length *n*, averaged over all messages and channel instances:

(33) P(e(C(n)≜1⌊enR⌋∑i=1⌊enR⌋E(WPrg(Y)≠I|Y(m,ℓℓ=1⌊enK⌋,X(m=x(mm=1⌊enR⌋,I=i,

where Y represents the random received vector, *I* is the random sent message, X(m represent random codewords equal to the codewords x(m of the given codebook C(n, while Y(m,ℓ are the random cloud vectors and the expectation E(W is taken according to the independent and identical conditional distribution *W*, generating the clouds Y(m,ℓℓ=1⌊enK⌋ given x(m. Let I(TV=minPA(TVP denote the mutual information of a pair of random variables with the joint distribution TV, and let us define:

(34) Ec(R,K)≜minPUD(U∥W|P)+|R−I(PU−|B(PU−K|+|+,

(35) E(e(R,K)≜maxPminU:I(PU+|B(PU−K|+≤RD(U∥W|P),

where *P* and *U* are such that U(y|x)P(x)≡T(y)V(x|y). Then, we can show the following.

**Theorem 2** (Converse bounds)**.** 
(36) lim infn→∞minC(n−1nlog1−P(e(C(n)≥E(c(R,K),

(37) lim supn→∞maxC(n−1nlogP(e(C(n)≤E(e(R,K),

*where (36) holds for all (R,K) and (37) holds a.e.: except possibly for such R(K) where there is a transition (a jump) from +∞ to a finite value of (35) as a monotonically non-increasing function of R.*

Let P(e(C(n|TV) denote the *conditional* average error probability of the maximum-likelihood decoder for a codebook C(n, given that the joint type of the sent and the received blocks is TV. Theorem 2 is a corollary of the following upper bound on the corresponding conditional probability of correct decoding:

**Lemma 8.**  *For any constant composition codebook C(n and any ϵ>0,*

(38) 1−P(e(C(n|TV)≤e−nR−I(TV−|B(TV−K|(+−ϵ+o(1),

*where the term o(1), vanishing uniformly w.r.t. TV as n→∞, depends on ϵ but does not depend on the choice of C(n.*

**Proof.**  Suppose we are given a constant composition codebook C(n, where all $\left\lfloor e^{nR}\right\rfloor$ codewords are of the same type with empirical probabilities P(x). Looking at the codebook C(n as a matrix of letters from X, of size $\lfloor e^{nR}\rfloor \times n$, we construct a whole ensemble of block codes by permuting the columns of the matrix. Observe that the total number of code permutations in the ensemble is given by

J≜enH(P+o(1)·π(P,

where H(P is the entropy of the empirical distribution *P*, and π(P denotes the number of same-symbol permutations in the type *P*, i.e., the symbol permutations that do not change a codeword that is a member of the type.

Suppose that, for each code in the ensemble, a separate independent channel instance is generated. And suppose that, for every transmission, one code in the ensemble (known to the decoder with its own channel instance) is chosen randomly with uniform probability over permutations. Consider an event where the sent codeword, chosen with uniform probability over the code permutations and the messages, together with the received vector have a joint type TV, such that $P\left(x\right)=\sum_{\;y}T\left(y\right)V\left(x\;|\;y\right)$. Since the channel-generating distribution is memoryless, this will result in the same conditional average probability of correct decoding given TV, when averaged over all messages and channel instances, as C(n itself. In what follows, we will derive an upper bound on this probability.

Let $\hat{y}$ be the received vector of the type *T*. Consider the conditional type class $T(V\;|\;\hat{y})$ of codewords with the empirical distribution *V* given the vector $\hat{y}$. Observe that the total number of all codewords in the ensemble belonging to this conditional type class (counted as distinct if corresponding to different code permutations or messages) is given by

(39) S≜enH(V|T+o(1)·π(P︸foramessagem·⌊enR⌋,

where H(V|T is the average entropy of the conditional distribution *V* given *T*, i.e., H(V|T=E(TV−logV(X|Y).

Let us fix two small numbers ϵ>δ>0 and consider separately two cases. Suppose, first, that K−B(TV≥ϵ. In this case, the probability of an event in which the cloud of any $x\in T(V\;|\;\hat{y})$ in the ensemble contains less than en(K−B(TV−δ) or more than en(K−B(TV+δ) vectors $\hat{y}$ by Lemma 2 uniformly tends to zero super-exponentially fast with *n*. Denote the complementary highly probable event as $\Omega (\hat{y})$. Let *k* be an index of a code in the ensemble. Let N(k) denote the number of codewords from the conditional type class $T(V\;|\;\hat{y})$ in the code of index *k*. Then, given the conditions that the received vector is $\hat{y}$, whereby the sent codeword belongs to $T(V\;|\;\hat{y})$, and $\Omega (\hat{y})$, we observe that the conditional probability of the code *k* is upper-bounded by $N\left(k\right)e^{2n\delta }/S$. Furthermore, given that indeed the code *k* is used for communication, the conditional probability of correct decoding is upper-bounded by $e^{2n\delta }/N\left(k\right)$. Summing over all codes, we can write

$$\begin{array}{cc} \mathrm{Pr}\left\{\mathrm{correct}\;\mathrm{decoding}\;|\;TV,\;\hat{y},\;\Omega \left(\hat{y}\right)\right\}\; & \leq \;\sum_{k:\;N(k)\;>\;0}\frac{N\left(k\right)e^{2n\delta }}{S}\cdot \frac{e^{2n\delta }}{N(k)}\;\;\leq \;\;J\cdot \frac{e^{4n\delta }}{S}\;\; \end{array}$$

(40) =e−nR−I(TV−4δ+o(1)

(41) ≤e−nR−I(TV−|B(TV−K|(+−5ϵ+o(1).

Consider now the second case when K−B(TV<ϵ. In this case, the probability of an event in which the cloud of any $x\in T(V\;|\;\hat{y})$ in the ensemble contains more than $e^{n(\epsilon \;+\;\delta )}$ occurrences of the vector $\hat{y}$ by (10) of Lemma 2 uniformly tends to zero super-exponentially fast. Let us denote this rare event as E(1(y^). In fact, among the codewords $x\in T(V\;|\;\hat{y})$, those with clouds containing $\hat{y}$ become rare. However, the probability of an event where, in the ensemble, there are less than S·e−n|B(TV−K|(++ϵ codewords from $T(V\;|\;\hat{y})$ having at least one vector $\hat{y}$ in their cloud uniformly tends to zero super-exponentially fast. This, in turn, can be verified similarly to (11) of Lemma 2, using (39). Let us denote this rare event as E(2(y^). Let us denote the complementary (to the union of the events E(1(y^) and E(2(y^)) and highly-probable event as $\tilde{\Omega }\left(\hat{y}\right)$.

Let $\tilde{N}\left(k\right)$ denote the number of such codewords in the code *k* that *both* belong to the conditional type class $T(V\;|\;\hat{y})$ *and* have at least one $\hat{y}$ in their respective cloud. Then, given the intersection of events that the received vector is $\hat{y}$ and that the sent codeword belongs to $T(V\;|\;\hat{y})$ and $\tilde{\Omega }\left(\hat{y}\right)$, we obtain that the conditional probability of the code *k* is upper-bounded by N˜(k)en|B(TV−K|(++2ϵ+δ/S. Given that the code *k* is used for communication, the conditional probability of correct decoding is upper-bounded by $e^{n(\epsilon \;+\;\delta )}/\tilde{N}\left(k\right)$. Repeating the steps leading to (40), we obtain (41) once again. □

**Proof of Theorem 2.**  First, we verify the bound on the correct-decoding exponent (36). It is enough to consider constant composition codes, because they can asymptotically achieve the same exponent of the correct-decoding probability as the best block codes, as is shown in the beginning of [7] [Lemma 5] using a suboptimal encoder–decoder pair.

Thus, let C(n be a constant composition codebook of a type *P*. Consider an event where the sent codeword together with the received vector have a joint type PU. The exponent of the probability of such event is given by D(U∥W|P)+o(1).

We then add the lower bound on the exponent of the conditional probability of correct decoding given PU of Lemma 8 in the following form:

(42) |R−I(PU−|B(PU−K|+|+−ϵ+o(1),

minimizing the resulting expression over all *distributions* PU, discarding o(1), and taking ϵ→0, we obtain (34).

Next, we establish the bound on the error exponent (37). Here, it also suffices to consider constant composition codebooks C(n, because there is only a polynomial number of different types in a general codebook of block length *n*.

Turning (38) into a lower bound on P(e(C(n|TV), we can obtain the following upper bound on the error exponent of C(n:

(43) maxP∈P(n(X)minU:PU∈P(n(X×Y),I(PU+|B(PU−K|+≤R−2ϵD(U∥W|P)+o(1)

(44) ≤maxP∈P(n(X)minU:I(PU+|B(PU−K|+≤R−3ϵD(U∥W|P)+o(1)

(45) ≤maxPminU:I(PU+|B(PU−K|+≤R−3ϵD(U∥W|P)+o(1).

Here, (43) follows directly from Lemma 8 and the fact that the exponent of PU is D(U∥W|P)+o(1). In (44), we extend the inner minimization from conditional types to arbitrary distributions *U* with the help of an additional ϵ in the minimization condition. In (45), we extend the outer maximization to arbitrary distributions *P*, and as a result, the maximum cannot decrease.

In the limit of a large *n*, the vanishing term o(1) in (45) disappears and we are left with ϵ. In order to replace ϵ>0 with zero, observe that both the objective function and the expression in the minimization condition of (45) are convex (∪) functions of *U*. It follows that the inner minimum of (45) is a convex (∪) function of ϵ∈R. Therefore, (45) itself, as a maximum of convex functions of ϵ, is convex (∪) in ϵ∈R. We conclude that by continuity of a convex function, the maximum in (45) tends to (35) as ϵ→0, with a possible exception when (45) jumps to +∞ exactly at ϵ=0, which corresponds to the jump to +∞ of (35) as a convex (∪) function of *R* exactly at *R*. □

## 5. Alternative Representation of the Converse Bounds

In this section, we develop alternative expressions for the converse bounds of Theorem 2. Using the properties that I(TV=minPA(TVP and ${\left|\;t\;\right|}^{+}=\max\;\left\{0,t\right\}$, the expression (34) for the lower bound of Theorem 2 can be written also as minPE(c(P,R,K), where

(46) E(c(P,R,K)≜minTVD(TV∥PW)+|R−A(TVP−|B(TVW−K|+|+,

and A(TVP and B(TVW are defined in (2). An alternative expression for (46) is given by

**Lemma 9** (Alternative representation—correct-decoding exponent)**.** 
(47) E(c(P,R,K)=min{sup0≤ρ<1E(0(−ρ,0,P)+ρR,sup0≤ρ<1E(0(−ρ,−ρ,P)+ρ(R+K)},

*where E(c(P,R,K) is defined by (46) and E(0 is defined as in (27).*

**Proof.**  We can rewrite (46) as a minimum of two terms:

min{minTVD(TV∥PW)+|R−A(TV|+,minTVD(TV∥PW)+|R−A(TV−B(TV+K|+}.

Solution of each one of the terms is similar to the method of Lemma 7 and gives (47). □

The expression (35) for the upper bound of Theorem 2 can be written alternatively as

**Lemma 10** (Alternative representation—upper bound on the error exponent)**.** 
(48) E(e(R,K)=maxPsup0≤η≤ρE(0(ρ,η,P)−ρR−ηK,

*where E(e(R,K) and E(0 are defined in (35) and (27), respectively.*

**Figure 1:**
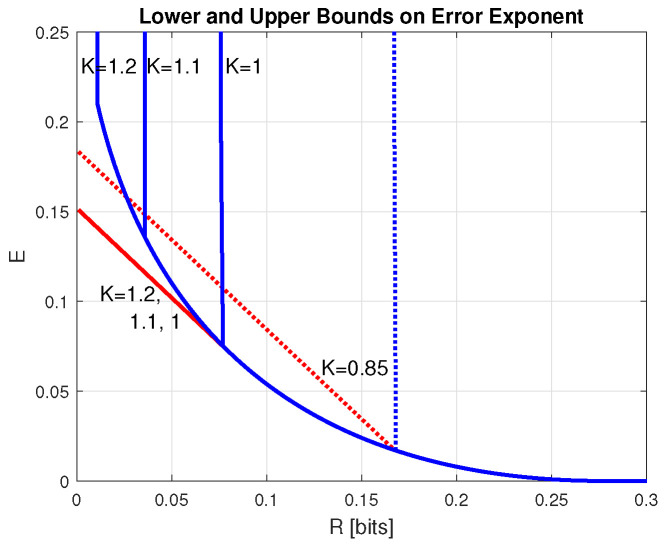
Lower and upper bounds on the optimal error exponent of the channel ensemble generated with W=BSC(0.2), as functions of *R*, for K=1.2,1.1,1, and 0.85. The lower and the upper bounds were obtained by (26) and (48), respectively, with P=(0.5,0.5).

The proof is given in the Appendix A. Examples of this bound together with the achievable error exponent as a lower bound are given in Figure 1. Note the discontinuities (jumps to +∞) in the upper bounds. Observing the alternative to (48) expression (A2), which appears in the proof of Lemma 10, it can be verified similarly to Lemma 7 that the discontinuity (jump to +∞) in (48) occurs at

R(min(K)=maxPminTVD(V∥P|T)+|B(TV−K|+=maxPsup0<β≤1−logmaxy∑xP(x)W(β(y|x)−βK.

For W=BSC(p), this gives R(min(K)=sup0<β≤1−log12(1−p)β+12pβ−βK, so that there is no jump for $K\geq -\frac{1}{2}\log\left[p(1-p)\right]$.

## 6. The Capacity of the Channel Ensemble

Let us define the capacity of the channel ensemble generated with *W*, denoted as C(W,K), as the supremum of rates *R*, for which there exists a sequence of codebooks C(n of size $\left\lfloor e^{nR}\right\rfloor$ with P(e(C(n)→0 as n→∞, where P(e(C(n) is defined as in (33). Comparing (1) with (33), we conclude that

P¯e(n)≥minC(nP(e(C(n).

**Figure 2:**
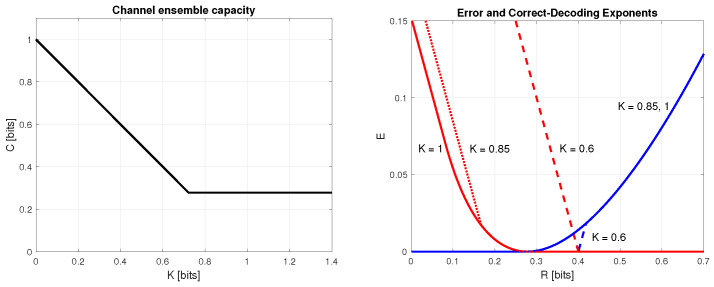
**Left graph**: The channel ensemble capacity C(W,K) vs. *K*, with W=BSC(0.2). **Right graph**: Achievable error exponents (decreasing curves) and converse correct-decoding exponents (increasing curves) as functions of *R*, for K=1, 0.85, 0.6, for the channel-generating distribution W=BSC(0.2). The curves were obtained by (26) and (47) with P=(0.5,0.5). Note that (47) for K=0.6 is not convex in *R*.

It follows that if the achievable error exponent (4) is positive, then there exists a sequence of codebooks C(n of size $\left\lfloor e^{nR}\right\rfloor$ such that P(e(C(n) drops to zero exponentially fast as n→∞. If, on the other hand, the lower bound (36) on the minimal correct-decoding exponent is positive, then for any sequence of codebooks C(n of size $\left\lfloor e^{nR}\right\rfloor$, the probability of correct decoding 1−P(e(C(n) tends to zero exponentially fast as n→∞, so that P(e(C(n) tends to 1. Then, C(W,K) must correspond to a point on the *R*-axis, at which both the maximal achievable error exponent and the lower bound on the minimal correct-decoding exponent of the channel ensemble are equal to zero. By the results of the previous sections, it turns out that there is only one such point. Examples are shown in Figure 2. We find the point C(W,K) in the following theorem.

**Theorem 3** (Ensemble capacity)**.**
C(W,K)=maxC(W),H(max(W)−K,

*where C(W) is the Shannon capacity of the DMC W, and H(max(W)≜maxPH(T with $\;T\left(y\right)=\sum_{\;x}P\left(x\right)W\left(y\;|\;x\right)$.*

**Proof.**  The maximal achievable error exponent, provided by Theorem 1, is

maxPE(e(P,R,K),

where E(e(P,R,K) is given by (3). The lower bound on the minimal correct-decoding exponent, given by Theorem 2, can be written as

minPE(c(P,R,K),

where E(c(P,R,K) is given by (46). Since D(TV∥PW)=0 if T(y)V(x|y)=P(x)W(y|x) for all (x,y), both expressions (3) and (46), as functions of *R*, meet zero at the same point, which is R=A(PWP+|B(PWW−K|+. This gives

C(W,K)=maxPA(PWP+|B(PWW−K|+=maxPmaxA(PWP,A(PWP+B(PWW−K=maxmaxPA(PWP,maxPA(PWP+B(PWW−K=maxC(W),H(max(W)−K,

where the last equality follows because A(PWP+B(PWW=H(T with $T\left(y\right)=\sum_{\;x}P\left(x\right)W\left(y\;|\;x\right)$, ∀y. □

## 7. The Optimal Correct-Decoding Exponent

In fact, the lower bound (36) is achievable. As in Section 3, suppose the codebook is generated i.i.d. according to a distribution over X with probabilities P(x), and let P(¯(e(n) denote the average error probability of the maximum-likelihood decoder, averaged over all possible messages, codebooks, and channel instances.

**Lemma 11** (Achievable correct-decoding exponent)**.** 
(49) lim supn→∞−1nlog1−P(¯(e(n)≤E(c(P,R,K),

*where E(c(P,R,K) is defined in (46).*

**Proof.**  Consider the following suboptimal decoder. The decoder works with a single anticipated joint type TV of the sent codeword x and the received vector $\hat{y}$. If the type of $\hat{y}$ is not *T*, the decoder declares an error. Otherwise, in case the type of the received block is indeed *T*, the decoder looks for the indices of the codewords with the conditional type *V* w.r.t. $\hat{y}$, with *at least one* replica of $\hat{y}$ in their clouds, and chooses one of these indices as its estimate $\hat{m}$ of the transmitted message. The choice is made randomly with uniform probability, regardless of the actual number of replicas of $\hat{y}$ in each cloud. If there are no codewords of the conditional type *V* w.r.t. $\hat{y}$ with at least one $\hat{y}$ in their cloud, then the decoder declares an error again.

Let N(nc denote the random number of *incorrect* codewords of the conditional type *V* w.r.t. $\hat{y}$, with at least one replica of $\hat{y}$ in their clouds, in the codebook. Then, the conditional probability of the correct decoding, given that the joint type of the received and the transmitted blocks is indeed TV, is given by

(50) E1N(nc+1≥1E[N(nc]+1,

with Jensen’s inequality where the expectation is w.r.t. the randomness of both the incorrect codewords and their clouds. Note that the exponent of E[N(nc] can be expressed as *R* minus (15) with TV. The RHS of (50) then results in the following upper bound on the exponent of the conditional probability of correct decoding:

(51) |R−A(TV−|B(TV−K|+|++o(1).

Adding to this the exponent of the joint type TV, we obtain (46). □

Now, since E(c(R,K)=minPE(c(P,R,K), by (36) of Theorem 2 and Lemma 11, we have the following.

**Theorem 4** (Optimal correct-decoding exponent)**.**
(52) limn→∞minC(n−1nlog1−P(e(C(n)=E(c(R,K),

*where E(c(R,K) is defined in (34) and P(e(C(n) is defined as in Section 4. This exponent is achievable by random coding.*

## Data Availability

The original contributions presented in this study are included in the article. Further inquiries can be directed to the corresponding authors.

## Abbreviations

The following abbreviations are used in this manuscript:

DMC	Discrete memoryless channel
RHS	Right-hand side
LHS	Left-hand side

## Appendix A

**Proof of Lemma 6.** The LHS of (24) equals 1 for α=1. Suppose α∈(0,q].

PrN≥N(1+1|N≥1=PrN≥N(1+1,N≥1PrN≥1=PrN≥N(1+1PrN≥1≥aPrN≥N(1+Z+1PrN≥1=bPrN≥N˜+1PrN≥1=cPrN≥N˜+1+PrN˜≥N+12PrN≥1=PrN≠N˜2PrN≥1=d12·1−p(2(0)−∑k=1Mp(2(k)1−p(0)=12·1+p(0)−∑k=1Mp(k)1−p(0)·p(k)≥12·1+p(0)−max1≤k≤Mp(k)≥12·1−max1≤k≤Mp(k)≥e12·1−maxe(112M2πMM−1,q(M,

where

(a) We add a nonnegative random variable Z∼Bernoulli(α), statistically independent with *N* and N(1.
(b) Random variable N˜=N(1+Z∼B(M,α) is statistically independent with *N*.
(c) We use the symmetry (i.i.d.) between $\tilde{N}$ and *N*.
(d) We denote $p\left(k\right)≜\mathrm{Pr}\left\{N=k\right\}$ and use the independence of $\tilde{N}$ and *N*.
(e) For k=M, we use p(M)≤q(M, and for 1≤k≤M−1, we use Stirling’s bounds [8] [Ch. II, Equation (9.15)] to obtain
  (A1) p(k)=Mkα(k(1−α)(M−k≤e(112M2πMk(M−k)e(−M·D(k/M∥α)≤e(112M2πMk(M−k)≤e(112M2πMM−1,
  where D(·∥·) is the binary Kullback–Leibler divergence.

□

**Proof of Lemma 10.** The proof can be obtained by a “sandwich” of the following inequalities:

(A2) maxPminP˜U:A(P˜UP+|B(P˜U−K|+≤RD(P˜U∥PW)

(A3) ≤amaxPminU:A(PUP+|B(PU−K|+≤RD(PU∥PW)

(A4) =bmaxPsup0≤η≤ρminUD(PU∥PW)+ρA(PUP+ηB(PU−ρR−ηK

=sup0≤η≤ρmaxPminUD(PU∥PW)+ρA(PUP+ηB(PU−ρR−ηK≤csup0≤η≤ρmaxPD(PU^(ρ,η∥PW)+ρA(PU^(ρ,ηP+ηB(PU^(ρ,η−ρR−ηK=dsup0≤η≤ρmaxPρE(PU^(ρ,ηlogc^−(1+ρ)E(PU^(ρ,ηlogc^(X)−ρDT∥T^(ρ,η−ρR−ηK=esup0≤η≤ρE(0ρ,η,P^(ρ,η−ρR−ηK=sup0≤η≤ρmaxPE(0(ρ,η,P)−ρR−ηK

(A5) =maxPsup0≤η≤ρE(0(ρ,η,P)−ρR−ηK,

where the transitions are as follows.

(a) The minimization over $\tilde{P}$ is removed by choosing $\tilde{P}\left(x\right)\equiv P\left(x\right)$.
(b) The equivalence between the minimum in (A3) and the supremum in (A4) can be shown by the method used in the proof of Lemma 7.
(c) The minimization over *U* is removed by choosing U(y|x)≡U^(ρ,η(y|x) for each pair (ρ,η), where U^(ρ,η is the conditional distribution derived and extended to all x∈X from the joint distribution (32):
  (A6) U^(ρ,η(y|x)≡1c^(x)·W1+η1+ρ(y|x)∑x˜P^(ρ,η(x˜)W1+η1+ρ(y|x˜)ρ,
  where $\hat{c}\left(x\right)$ is the normalizing constant for each *x* and P^(ρ,η is a special input distribution achieving the maximum of (27):
  P^(ρ,η∈argmaxPE(0(ρ,η,P).
  Note that the conditional distribution U^(ρ,η is well defined (adds up to 1) for *all* x∈X, i.e., $\hat{c}\left(x\right)>0,\forall x$. This is because the normalizing constant $\hat{c}\left(x\right)$ is proportional to the derivative of the expression inside log of (27) w.r.t. P(x) at P^(ρ,η(x), and therefore, as in [9] [Equations (22) and (23)], it must *necessarily* satisfy
  c^(x)=c^,P^(ρ,η(x)>0,c^(x)≥c^,P^(ρ,η(x)=0.
  Otherwise, it would be possible to redistribute the probability mass P^(ρ,η(x) to further increase (27).
(d) The objective function is rewritten with c^=∑xP^(ρ,η(x)c^(x), T(y)≡∑xP(x)U^(ρ,η(y|x), and T^(ρ,η≫U^(ρ,η(·|x), ∀x∈X, which is the marginal distribution of *y* derived from the joint distribution (32) with P^(ρ,η:
  (A7) T^(ρ,η(y)≡1c^·∑xP^(ρ,η(x)W1+η1+ρ(y|x)1+ρ.
(e) Observe that any distribution *P* maximizes E(PU^(ρ,ηlogc^, while any distribution P≪P^(ρ,η maximizes E(PU^(ρ,η−logc^(X). Ultimately, the choice P=P^(ρ,η gives DT∥T^(ρ,η=0, maximizing the whole expression, where E(0ρ,η,P^(ρ,η=−logc^.

Finally, observe that (A3) is the same as (35). The equivalence of the minimum in (A2) and the supremum in (A5) can be shown as in the proof of Lemma 7. □
